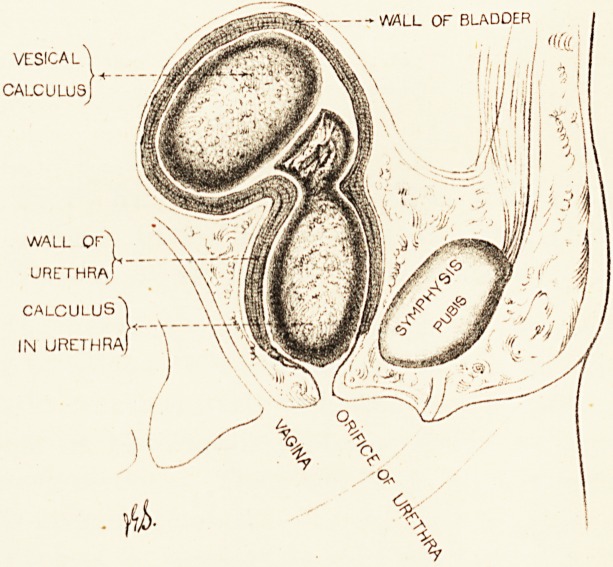# Some Interesting Cases of Stone in the Bladder

**Published:** 1887-09

**Authors:** J. Greig Smith

**Affiliations:** Surgeon to the Bristol Royal Infirmary


### Clinical Records.

---

### SOME INTERESTING CASES OF STONE IN THE BLADDER. By J. Greig Smith, M.A., F.R.S.E., Surgeon to the Bristol Royal Infirmary.

*(Read at a meeting of the Bristol Medico-Chirurgical Society.)*

### REMOVAL OF A STONE WEIGHING OVER NINE OUNCES BY THE SUPRA-PUBIC METHOD IN A PATIENT WITH SUPPURATIVE NEPHRITIS AND DISCHARGING EMPYEMA. DEATH ON THE TENTH DAY.

J. T., æt. 58, was sent to me with what was supposed to be caries of the ribs. His history was, that for ten years he had been more or less of an invalid, but that six months previously he had been taken seriously ill with a pulmonary trouble. After being very ill for some weeks, with great pain on the right side and difficult breathing, he suddenly coughed up large quantities of pus, and then he improved a little. Soon after, an abscess burst between the 7th and 8th ribs in the axillary line; and the sinus had continued open ever since, discharging large quantities of thick pus.

When I first saw him, he was so much exhausted after a long journey that a minute examination was impossible. However, the existence of dulness, hardness, and some œdema in the right loin, between the crest of the ilium and the ribs, made me look to the kidney. I diagnosed suppurative nephritis : the pus having escaped from the kidney-capsule, burrowed its way upwards through the diaphragm into the pleura, and ultimately having burst through the lung. This diagnosis I communicated to his medical attendant. Pus in the urine, and frequent micturition, of which he informed me only after direct questioning, I thought might be accounted for in this way.

For a few days further examination was impossible, as he continued very weak and ill. A probe entered the sinus upwards for eight inches, and could be moved freely in all directions in the pleural cavity. I inserted a drainage tube, and put the patient on stimulating diet.

Questioned as to the possibility of stone in the bladder, he told me that he had had what his medical advisers (and he had had many) called an irritable bladder; that he had been frequently sounded, and no stone found; and that quite recently one of his medical men had been in the habit of washing out his bladder. He was certain there was no stone, and he begged me not to pass an instrument, as it gave him so much pain. He had never noticed blood in the urine.

The finger in the rectum soon settled the diagnosis. The first thing felt was a large stone, lying almost like a polypus in the rectum, and blocking its calibre. The bladder was evidently drawn out like a Florence flask, the stone occupying its fundus. A short-beaked sound, introduced point backwards and tilted upwards—in the directions reverse to the ordinary method—came in contact with the stone, but could not measure its size, on account of the contracted state of the bladder.

The diagnosis was now, that the stone had set up the kidney trouble—that it was a backward nephritis. And, under all the circumstances, I refused to operate. I explained to him that the lithotomy alone would be a very serious operation for a man in fair condition, but that in his case it would almost certainly be fatal. And when the stone was removed, the suppurating kidney would require further operation ; while as to the empyema, that also was a serious condition. I further explained that the administration of an anæsthetic to a man whose lungs were not sound was attended with risk.

The patient was not strong enough to return to his home, and he continued under my care. He had little sleep, being waked up every half-hour or less to micturate, and being kept awake for some time by the pain which followed micturition. At every visit he urged me to operate, if it were only to relieve him of his agony and give him a few hours' consecutive sleep. His medical man advised operation, and a friend and colleague who gave me the benefit of his opinion thought it right to operate. On October 12th, I therefore operated by the supra-pubic method.

The bladder was first washed out with boro-glyceride solution by means of an irrigator, the catheter being left in, attached to the irrigator tube. The rectal bag was then inserted, and distended with water. The ordinary dissection was rapidly made, and the recti partially divided near their insertion. When the bladder was exposed, the irrigation-reservoir was elevated a few feet, and the bladder was rapidly distended, while the peritoneal fold could be felt and seen to be carried upwards. When distension was sufficient, the irrigator was simply placed on the level of the bladder, ligation of the penis being quite unnecessary. A Lister's sinus-forceps was gently insinuated through the coats of the bladder, and separated them ; while by pulling the bladder-walls forward, they gave easy opportunity for the placing of two catch forceps at the sides of the opening. The opening was then enlarged, chiefly downwards, by scissors. The stone was first tilted on its side by the finger, and then extracted by long flat-bladed lithotomy forceps.

The wound in the bladder was attached to the upper extremity of the abdominal wound ; bladder-wall, muscles, skin, and fascia being gathered together in the same sutures. It was not possible, on account of the extreme friability of the vesical tissue, to satisfactorily unite the wall of the viscus to the abdominal wound in its lower portion. A large drainage tube was placed in the bladder, and the patient laid on his side. The interior of the bladder was rubbed over with boro-glyceride, and the edges of the wound and neighbouring skin freely smeared with the pure material.

When the patient was first seen, a few hours afterwards, he was breathing with great difficulty, bronchial râles being audible all over the room, and for the first twenty-four hours his lungs seemed to get steadily more and more œdematous. Then, with the help of strong stimulating expectorants, he began to get up a little mucus, and for the first four days he greatly improved, sleeping almost constantly, and expressing himself as being delighted with the relief afforded. But about the sixth day the expectoration somewhat suddenly became more abundant and quite purulent. I judged that the old communication with the lung had re-opened. He could breathe only in the sitting position. In spite of every therapeutic means, his lungs got more and more laden with purulent material, and he died asphyxiated.

The wound had progressed most satisfactorily, never showing a sign of redness, and healing perfectly, all except a small sinus, through which clear urine trickled. He had begun to pass a little through the penis. It pained him scarcely at all, even during his prolonged fits of coughing. A better result, so far as the operation was concerned, could scarcely have been wished for; it certainly was not expected.

After the operation, the sinus in the side discharged less freely, in spite of free drainage; this also was in favour of the re-opening of the old pulmonary fistula. A *post-mortem* examination was not held.

This was the fifth occasion on which I had performed supra-pubic lithotomy; and nothing has impressed me so much with the excellence of this method as the local result in this case. Considering the size of the stone, and the nature of the enormously thickened and non-distensile bladder walls, the proceeding was peculiarly easy and rapid. In any case, it was the only feasible method. Crushing was out of the question, and perineal lithotomy, without crushing, would have been impossible.

*Remarks.*—I dare now to say in public, what I have always said in private, that perineal lithotomy is, in my opinion, a grand surgical error.

Ever since I have had an opportunity of performing a major operation, I have spoken against perineal lithotomy. A surgeon to a large general hospital for seven years, I have never operated for stone by perineal section. In every case where crushing was out of the question—and such cases are very few—I have performed the supra-pubic operation. This opinion I held before Bigelow came on the field: his advent has confirmed my practice.

I have often heard an excellent surgeon, now dead, and much respected and admired by many of us, say that he had often to finish a colleague's lithotomies by curing their strictures or their fistulæ. Such sequences of lithotomy somehow seemed to gravitate towards him: he put it down to the mode of operating by the median method. Very possibly some one else will have to treat his cases done by the lateral method. It is certain that in each case the operations were as brilliantly performed, and the immediate results were as good, as surgical science and art could bring about. And yet only a few months ago, in our Infirmary, a colleague had to treat a case of perineal fistula (fortunately with success) for a patient on whom lithotomy had been performed by one of these surgeons some ten years ago. In my own comparatively limited experience, I have seen three cases of fistula, and four of urethral stricture, all following perineal lithotomy.

It is undoubtedly true that stricture, fistula, and sexual incompetence are by no means rare sequences of perineal lithotomy. A London hospital surgeon has recently said that in 200 cases in which he had performed perineal lithotomy, he had never known any of these results follow. I would venture to affirm that a written statement from each of these patients would give very different information. That the seminal ducts may be divided, and the urethra be cut, bruised, or torn, with impunity, is dead against all that we know of pathology, and physiology as well.

It was not, however, a consideration of these remote results which weighed so much with me, as the plain, palpable, straightforward fact, that in lateral lithotomy we sought a devious and dubious, not to say hap-hazard, mode of entrance into the bladder, when we had provided a simple and straightforward one over the pubes. If the results from the high operation were worse than the results from the low, then I felt certain that this was because of some error in technique. If only one tithe of the anatomical study and description which has been spent on the perineum had been spent on the supra-pubic space, the result might have been different. It was terror of the peritoneum, probably, which drove surgeons from the supra-pubic operation. In respect of the operation in question, Lister was the first to dispel this terror; to him, without a doubt, must be awarded the merit of having re-introduced the supra-pubic method of lithotomy.

One of my most treasured possessions is a copy, dated 1722, of Cheselden's *Treatise on the High Operation for Stone.* To his abandonment of this operation, I trace the resumption of the lateral method by others. "Cutting on the Gripe," as the old Roman and Greek operation was called; dignified into a surgical procedure by the major operation of Marian in 1524; degenerated into semi-quackery by Frère Jacques in 1697, was, in its anatomical outcome, lateral lithotomy. Cheselden's fresh young ideas were theoretically correct; where he failed in practice, from ignorance of now ascertained principles of surgery, he took the intelligible course of following on a sound anatomical basis the palpably successful practice of ancients and quacks. The real inventor of the operation, Pierre Franco (1561), successful as he was, did not advise any one to follow him. Cheselden himself even, after writing such a treatise, and with his (for those days) complete knowledge of anatomy, abandoned it for the lateral method. And herein he followed his lights: he knew nothing of the germ theory; his vulnerary decoctions were aseptic, but not antiseptic; he did not know how to avoid the peritoneum in operating; and he knew not how to protect the supra-pubic wound in the after-treatment. Had he known these things, he would never have abandoned the practice of those years when his intellect was at its best, and his hands were most skilful.

Anatomy rose in the schools, and the perineum was dissected and described in all its intricacies of muscles, fasciæ, arteries, and nerves. But all this dissection and description could never make lateral lithotomy more than a stab in the dark, or at best a cutting by rule of thumb —or, if you like, by guidance of staff and forefinger. Now this is fortuitous surgery. True, in children at least, the chances have been highly favourable, the recoveries have been numerous, and the immediate results favourable. But we ought to take congnisance of remote results. And this is now being done. Incompetence, stricture, and even perineal fistula, must be counted among the possible results of lateral lithotomy.

In the supra-pubic operation, the recovering patient is well, not only after the operation, but for ever afterwards. He has nothing to dread. The seminal ducts are intact, the membranous urethra is undivided. The bladder-scar is in an area remote from urethra and seminal duct, and it causes no harm whatever. If its immediate mortality at all closely approaches that of the perineal operation, the supra-pubic is the preferable. At present it is quite as good, and the rapidity of recovery is greater. In the future I have no doubt whatever that, where crushing and immediate evacuation of the stone is out of court, supra-pubic lithotomy will uniformly take the place of the perineal operation.

In the operation of supra-pubic lithotomy, I think we have made in the last few years of its revival several notable advances. Foremost among these I would place suturing of the bladder in children. In quite a considerable number of cases primary union has been obtained, and in the great majority union has been complete within three weeks. In old people, and in cases of very large stones, supra-pubic drainage is, I think, advisable. But, even in these, healing will be accelerated by partially suturing the opening; and more particularly by suturing the bladder-opening in the stitches which close the abdominal wound.

The second advance is the rejection of the catheter placed in the urethra. I am sure that twice in young patients of mine primary union was prevented by over-distension of the bladder, caused by blocking of the catheter during the night. But the fistula which formed was small, and healed up more quickly than does the perineal wound in lateral lithotomy. I had several times discussed the advisability of doing without the catheter, and had decided to try the experiment, when Mr. Barker's success solved its feasibility. Other cases have proved the wisdom of his recommendation—one of them in my own practice.

As to the rectal bag, my mind is not made up. In the removal of a very large stone I do not think I should again use it, but should prefer instead the fingers of an assistant in the rectum, which would push the stone up to, or out of, the wound. And in such cases the bladder tissues are so friable that rectal distension might damage or even tear them. In children the bladder lies naturally high, and I doubt if the rectal bag does much good. It steadies the parts, and, when the bladder is opened, it keeps forward the posterior wall, and brings it within easy reach of the finger. In the removal of bladder-tumours I have found that, on these grounds, the rectal bag is very useful.

What has usually been claimed as the chief advantage of the rectal bag, namely, increasing of the supra-pubic interval, I should regard as the least. A little experience of peritoneal surgery soon teaches us how to recognise and push up that membrane in epicystotomy. The advantage of increasing this interval, at least, is only momentary: when the bladder collapses, after being opened, the increase of the interval is lost. Then the whole end may be attained by pushing the peritoneum inwards on the abdominal cavity.

---

### CASE OF CALCULUS IN URETHRA WITH CALCULUS IN BLADDER IN A GIRL EIGHT YEARS OLD.

The patient, a girl of eight, but small for her years, was admitted to the Bristol Royal Infirmary on Jan. 8th, 1887, suffering from incontinence of urine. Every quarter of an hour or so she tried to pass water, but most of it dribbled away in the intervals. There was considerable pain, with marked tenesmus. The urine was often stained with blood, and blood was seen in the evacuations. On Jan. 14th the child was put under the influence of chloroform. A probe passed into the bladder struck a stone of considerable dimensions. It was noted at the time that the urethra was long and rough, apparently coated with phosphates, but urethral calculus was not diagnosed.

On Jan. 17th I proceeded to remove the calculus through the urethra. A small incision was made by scissors on the under surface of the urethral orifice, and the canal behind was dilated with a Lister's sinus-forceps. During dilatation I was surprised to find a stone in contiguity with the forceps, which was at once and easily extracted. I concluded that this was the stone in the bladder, and that the urethra was very short. Its curious shape (as shown in the drawing, made to scale) and the existence of a facet on its surface suggested the existence of another stone. I pushed my little finger, which was as large as the parts would admit without risk of laceration, and was able to feel at its tip a small opening: a probe pushed through this opening at once came upon a stone which was evidently closely embraced by the walls of the bladder, as in a saccule. I cautiously dilated this opening with the finger sufficiently to admit the introduction of a polypus-forceps, and by conjoined manipulation through rectum and over pubes endeavoured to place the stone between the blades. After several ineffectual attempts the stone was caught, but it could not be extracted through the small opening without tearing it. I therefore crushed it with a small lithotrite, and proceeded to remove it piecemeal. This proved to be a difficult and delicate proceeding. The sac in which the stone lay (in reality the contracted bladder) was not in a line with the urethra, but behind and below it; no instrument could be found with a curve sharp enough to enter it through the urethra. Washing out the fragments with a Bigelow's apparatus was also found impracticable. Most of the fragments were removed between the tip of the little finger and a small scoop, while a finger in the rectum pushed the bladder forwards. The smallest fragments were finally washed out. The very small size of the parts, and their delicacy and friability, made it imperative that all proceedings should be carried out with extreme gentleness.

The patient took the anæsthetic very badly; shortly after the commencement of the operation she had a well-marked epileptic fit.

The patient made an uneventful recovery. By Feb. 2nd she had recovered complete control over the bladder, and three days later she was discharged well.

*Remarks.*—Not till the operation was over and the calculi carefully examined did it strike me that this was a case of combined urethral and vesical calculus. The shape of the calculus first removed, and the polished facet on the knob which sprang from its inner extremity, showed that it had been partly encapsuled, and that a second stone rubbed against the knob which protruded from the saccule on which it rested. That this stone lay in the urethra I have now no doubt. It lay quite close to the orifice of the urethra; and the urethral canal had been gradually distended and elongated by the growing stone, so that the neck of the bladder was pushed some distance backwards. It was coated with epithelial *débris* and mucus, and the probe first used in sounding the stone glided easily over it without producing the characteristic sensation.

So firmly was the bladder contracted around the second stone that sacculation was at first diagnosed. The stone could be readily palpated between the finger in the rectum and the hand over the pubes, but from the continued straining and struggling of the child (profound anæsthesia at once induced alarming symptoms) it slipped from the grasp in the most aggravating way. And when forceps had gripped it, it was simply impossible to pull it through the narrow orifice at the neck of

WALL OF BLADDER

VESICAL CALCULUS

WALL OF URETHRA

CALCULUS IN URETHRA

SYMPHYSIS PUBIS

VAGINA

ORIFICE OF URETHRA

the bladder, dilated as it had been by the finger. It was necessary to crush; and to remove the fragments one by one through the narrow opening without causing injury to the delicate tissues, was one of the most tedious and difficult surgical operations in which I have been engaged, Much time was wasted in attending to the dangerous symptoms arising from the anæsthesia; the whole operation occupied the greater part of an hour.

---

*(To be continued.)*

---

## Figures and Tables

**Figure f1:**